# How should clinicians assess acute dental pain?: A review

**DOI:** 10.1097/MD.0000000000031727

**Published:** 2022-11-11

**Authors:** Shinpei Matsuda, Hayato Itoi, Takashi Ryoke, Hitoshi Yoshimura

**Affiliations:** a Department of Dentistry and Oral Surgery, Unit of Sensory and Locomotor Medicine, Division of Medicine, Faculty of Medical Sciences University of Fukui Fukui Japan.

**Keywords:** acute pain, dental, head and neck regions, pain assessment

## Abstract

Pain is the most common complaint in the dental field and may have a significant impact on the patients’ quality of life. However, objective pain assessment is sometimes difficult, and medical and dental clinicians may encounter cases of pain in the head and neck region, making it difficult to establish differential diagnoses. This study aimed to review acute pain in clinical dentistry at each phase of dental procedures and discuss the current status and issues in the development of acute dental pain assessment methods in the future. Acute pain in clinical dentistry may differ in nature and modifying conditions of pain at each stage: before dental procedures, while visiting dentists, and during and after dental procedures. They are related to actual or potential tissue damage, and may be modified and aided by personal experiences, including psychological and social factors. With respect to the aging and multinational population and pandemic of infectious diseases, significant breakthroughs in the development of new pain scales without verbal descriptions are desirable. Furthermore, it is expected that a new pain scale that can be applied to acute pain in the head and neck regions, including the oral cavity, will be developed.

## 1. Introduction

Pain, including acute and chronic, is the most common complaint in the dental field and may have a significant impact on patients’ quality of life.^[1,2]^ The nature and characteristics of these pains are useful for clinical diagnosis.^[1,2]^ On the other hand, objective pain assessment is sometimes difficult, and medical and dental clinicians may encounter cases with pain in the head and neck region, making it difficult to make differential diagnoses, including dental infections and inflammatory diseases, temporomandibular disorders, headaches, trigeminal neuropathy associated pain, and idiopathic or centralized pain conditions.^[1,2]^

Acute pain can occur in the hard and soft tissues of the mouth in patients of all ages.^[3,4]^ Patients with pain sometimes need to visit the emergency department and receive analgesics.^[3–5]^ The American Dental Association recommended that the selection of oral analgesics and nonsteroidal anti-inflammatory drugs as the first-line therapy for acute pain management depends on the degree of expected pain classified into 4 levels as follows: mild, mild to moderate, moderate to severe, and severe.^[4,6–8]^ This recommendation was adopted based on studies conducted on postprocedural acute pain.^[4,6–8]^ In addition, this recommendation suggests that the management of acute dental pain should be predicted and classified according to the type of dental intervention.^[4,6–8]^

In July 2020, the International Association for the Study of Pain (IASP) announced a change in the definition of pain as follows; “*An unpleasant sensory and emotional experience associated with, or resembling that associated with, actual or potential tissue damage*,” from the IASP statement in 1979 as “*An unpleasant sensory and emotional experience associated with actual or potential tissue damage, or described in terms of such damage*.”^[9,10]^ They then appended the detailed statement that pain is a personal experience influenced by biological, psychological, and social factors, and verbal description is only one of several behaviors to express pain.^[9,10]^ This announcement was very important for medical or dental professionals involved in clinical practice and researchers investigating pain.

In May 2021, the World Health Organization (WHO) adopted World Health Assembly (WHA)74.5 suggesting the urgent need for improved oral hygiene worldwide.^[11–13]^ Based on the 17 Sustainable Development Goals in the 2030 Agenda for Sustainable Development, WHA74.5 recommends recognizing the importance of Goal 3, and interrelationships between oral health and other sustainable development goals as follows: Goal 1, Goal 2, Goal 4, and Goal 12.^[11,13,14]^ Poor oral health is associated with pain and affects quality of life and social activities.^[11,12]^ Researchers may need to reconsider “pain” in the dental field based on the IASP and WHO statements.^[9–12]^

In the dental field, the authors were concerned that acute pain was studied less than chronic pain, including orofacial pain and temporomandibular disorders. In addition, the authors were concerned that recent clinical research in the dental field, including oral health and pain research, has not progressed in relation to the coronavirus disease 2019 (COVID-2019) pandemic.^[15,16]^

This study aimed to review acute pain in clinical dentistry at each phase of dental procedures and to discuss the current status and issues in the development of acute dental pain assessment methods in the future, based on the change in the definition of pain announced by the IASP in 2020.^[9,10]^

## 2. Acute pain in clinical dental practice

### 2.1. Before dental procedure

Acute hard tissue pain in the dental field can occur due to infectious and inflammatory dental diseases such as dental caries, pulpitis, gingivitis, periodontitis, jaw inflammation, and odontogenic maxillary sinusitis.^[17,18]^ In trauma-related cases, pulpitis, fractured teeth, fractures of the jaw or alveolar bone, and temporomandibular joint damage-related pain must also be considered.^[2,19–21]^

The most common type of acute soft tissue pain in dentistry is stomatitis.^[22]^ In trauma-related cases, soft tissue damage and injury should be considered.^[21]^ salivary gland-related disorders, with or without infection, may also present as acute soft tissue pain.^[23]^

Considering the definition of pain announced by the IASP in 2020, acute dental pain, including toothache, may be modified by personal experiences such as mechanical hypersensitivity.^[9,10,24]^

### 2.2. Visiting a dentist and during dental procedure

Dental fear and anxiety are not rare conditions, and patients visiting a dentist may be nervous based on their personal experience.^[25,26]^ Previous systematic review reported that the prevalence of dental fear and anxiety was approximately 10%.^[26]^ A close association between them and pain sensitivity has been suggested.^[25–27]^ In addition, the sound of dental treatment devices, including dental turbines, engines, and scalers may affect autonomic responses, such as cerebral blood flow and metabolism, and may induce pain associated with dental fear and anxiety.^[25–29]^ Furthermore, dental patients are exposed to uncertainty and vulnerability regarding their future treatment.^[30]^

During dental procedures, acute pain related to the injection of dental local anesthesia with actual tissue damage occurs with or without dental surface anesthesia.^[31,32]^ In addition, the sound of instruments and devices, as well as damage and pressure to hard and soft tissues, cannot be completely eliminated. Thus, it is difficult for dental procedures to completely eliminate pain via the trigeminal nervous system and pain associated with the 5 senses and personal experience, including dental fear and anxiety.^[25–29,33–35]^

Based on the IASP statement in 2020, such as pain is mediated by “*actual or potential tissue damage*” and “*a personal experience influenced by psychological and social factors*,” previous reports seem to support that patients’ subjective acute dental pain may be modified and triggered while visiting dentists and during dental procedures.^[9,10]^

### 2.3. After dental procedure

Tissue damage associated with surgical and nonsurgical dental procedures often causes acute pain. Acute dental pain after dental procedures has been advocated to be managed by pre-procedure analgesia, cold compression, use of long-acting anesthetics, and drug therapy.^[3–8,36]^ Drug therapies, including acetaminophen, nonsteroidal anti-inflammatory drugs, and opioids, for acute dental pain are controversial because of their side effects.^[3,4,16,37]^ Acute dental pain resolves after healing of the damaged tissue or after routine dental procedures; however, pain persists and develops into chronic pain in a few cases.^[20]^

Based on the IASP statement in 2020, the application of appropriate postoperative analgesic methods for acute dental pain caused by “*actual or potential tissue damage*” is important to prevent the transition to chronic pain.^[9,10]^

## 3. Discussion

This review summarizes acute pain that should be considered in clinical dental practice, considering the definition of pain announced by the IASP in 2020 at each phase as follows: before dental procedures, visiting a dentist and during dental procedures, and after dental procedures.^[9,10]^ Dentists need to properly assess acute dental pain by considering sensory, emotional, and personal experiences as well as hard and soft tissue damage.^[9,10]^

Acute dental pain is caused by infectious or inflammatory dental disease and dental intervention. The biological characteristics of acute pain have been reported to depend upon its stimulation, which can be divided into 2 main mechanisms: peripheral and central stimulatory responses.^[17]^ In dentistry, appropriate treatment, including endodontics and surgical intervention, is the first priority for acute dental pain. For these reasons, the authors considered the possibility that clinical research on the assessment of acute dental pain might be inadequate compared to chronic pain. However, during dental procedures with acute pain, blood pressure fluctuations and vasovagal reflexes may occur because of changes in the autonomic nervous system.^[38,39]^ These conditions should be assessed and managed by dentists before, during, and after dental treatment. However, these are still important dental clinical problems because they are complex situations related to the subjective psychological state of the patient and acute dental pain.^[38,39]^ In light of this point of view, the authors listed and discussed the current status and issues in the development of acute dental pain assessment methods.

### 3.1. Development of objective acute dental pain assessment methods

Objective pain assessment is challenging in clinical dentistry. Although many studies have been conducted, most have focused on chronic pain, and there have been few studies on the head and neck region.^[40]^ Visual analog scale (VAS) has often been used to assess pain related to dental procedure.^[32,41]^ VAS was first published in its original form about 100 years ago in the psychological field and has been used as the “gold standard” method of objective assessment in various fields.^[41,42]^ VAS is also important in recent studies on objective methods for assessing pain in the head and neck region, and no significant breakthroughs have been found in this area.^[43,44]^ There are significant individual differences in the expression of pain-related images.^[45]^ Therefore, it is necessary to present the participants with a picture, diagram, or graphic of the pain in advance to objectively and quantitatively assess subjective pain.^[46]^ The authors hope that breakthroughs will be made in the research area of acute pain assessment methods to replace the VAS, which has been in use for nearly 100 years.

### 3.2. Development of acute dental pain assessment methods without verbal description

Many verbal expressions and multiple questionnaires may be added to the pain assessment methodology to account for the subject’s personal experience, which is influenced by biological, psychological, and social factors.^[44,47,48]^ In this aspect, many pain studies may run counter the 2020 IASP statement that “verbal description is only one of several behaviors to express pain.”^[9,10]^ Furthermore, as exemplified by the Bouba/kiki effect, the image evoked by the word “Pain” may be affected by the native and non-native English speakers.^[49]^ Based on the statement of a change in the definition of pain by IASP in 2020, WHO statement, and experience with the COVID-19 pandemic, it is hoped that universal and nonverbal pain assessment methods will be proposed.^[9–13,15,16,48]^ In the future, it is hoped that new acute dental pain assessment methods without verbal descriptions will be researched and developed.

### 3.3. Development of acute dental pain assessment methods using medical and scientific technology

Recently, artificial intelligence has been applied to facial identification and has been considered to ensure objectivity in the assessment of pain.^[50–52]^ On the other hand, they may require a large amount of appropriate training materials, and ethical issues of data collection and bias in data selection may intervene.^[50–52]^ In addition, studies of acute pain assessment using functional magnetic resonance imaging have been reported in recent years.^[53,54]^ Currently, these scientific and medical technologies are still in the research phase and their application in clinical dentistry may not be realistic. The authors believe that these methods are at a stage where ethical issues need to be resolved and evidence collected.

Considering the change in the definition of pain announced by the IASP in 2020, this narrative review provided detailed conditions and circumstances of acute pain in each phase of clinical dental practice.^[9,10,55,56]^ Furthermore, this study revealed that many issues must be resolved in the development of new assessment methods for acute dental pain. With respect to the global aging and multinational population and infectious diseases affecting the respiratory system, such as the COVID-2019 pandemic, significant breakthroughs in the development of new pain scales without verbal descriptions are desirable for achieving WHA74.5, published by the WHO.^[11]^

## 4. Conclusion

In this review, the authors considered the urgent need for the development and clinical application of a new objective and universal pain scale without verbal descriptions, considering global problems such as the aging of the population, multinationalization, and the pandemic of infectious diseases. The authors hope that significant breakthroughs will be made in the development of a new pain scale that can be applied to acute pain in clinical dentistry.

## Author contributions

**Conceptualization:** Shinpei Matsuda.

**Writing – original draft:** Shinpei Matsuda.

**Writing – review & editing:** Shinpei Matsuda, Hayato Itoi, Takashi Ryoke, Hitoshi Yoshimura.
